# Variations in Postpartum Readmission by Individual Race, Ethnicity, and Rurality in South Carolina

**DOI:** 10.1001/jamanetworkopen.2025.47455

**Published:** 2025-12-08

**Authors:** Curisa M. Tucker, Yunqing Ma, Jiajia Zhang, Md. Utba Rashid, Jihong Liu, Berry Campbell, Xiaoming Li, Peiyin Hung

**Affiliations:** 1 Department of Biobehavioral Health & Nursing Science, College of Nursing, University of South Carolina, Columbia; 2 Department of Epidemiology and Biostatistics, Arnold School of Public Health, University of South Carolina, Columbia; 3 Obstetrics and Gynecology, School of Medicine Columbia, University of South Carolina, Columbia; 4 Health Promotion, Education, and Behavior, Arnold School of Public Health, University of South Carolina, Columbia

## Abstract

**Importance:**

Racial and ethnic disparities in postpartum readmission (PPR) remain a critical public health concern, with non-Hispanic Black individuals experiencing rates up to 3 times as high as non-Hispanic White individuals. PPR is often associated with mental health disorders (MHDs) and substance use disorders (SUDs), and barriers to care are particularly acute in rural communities.

**Objective:**

To investigate intersections in the associations of individual residential rurality and race and ethnicity with all-cause, MHD-, and SUD-related PPR throughout 1 year post partum.

**Design, Setting, and Participants:**

This statewide retrospective cohort study used data from birth certificates linked to all-payer hospital data for individuals 15 to 50 years of age who gave birth and were discharged from South Carolina hospitals between January 1, 2018, and December 31, 2021. Data analyses were completed August 17, 2025.

**Exposures:**

Individual race and ethnicity and individual residential location at time of birth.

**Main Outcomes and Measures:**

Cumulative incidence of all-cause, MHD-, and SUD-related PPR at 42, 90, 180, and 365 days post partum. Cox proportional hazards models estimated adjusted hazard ratios (AHRs) and 95% CIs for 1-year follow-up, adjusting for individual characteristics.

**Results:**

Of 190 645 births to 166 330 unique individuals (mean [SD] age, 28.2 [5.8] years; 4.9% Hispanic, 30.9% non-Hispanic Black, and 57.1% non-Hispanic White), the highest percentage (30.4%) included individuals between 25 and 29 years of age; 27 961 (14.7%) of births were to women residing in rural areas and 162 684 (85.3%) of births were to women residing in urban areas. Up to 1 year post partum, 4.7% of birthing individuals had all-cause PPR, 1.5% had MHD-related PPR, and 0.8% had SUD-related PPR. In adjusted models, non-Hispanic Black individuals had higher risk of all-cause PPR compared with non-Hispanic White individuals in urban areas (AHR, 1.38 [95% CI, 1.31-1.45]), whereas Hispanic individuals had lower risk (AHR, 0.83 [95% CI, 0.74-0.93]). Rural residence was associated with increased all-cause PPR risk overall (AHR, 1.15 [95% CI, 1.06-1.25]) but was also associated with reduced racial and ethnic disparities in all-cause PPR (interaction AHR, 0.86 [95% CI, 0.77-0.97] for non-Hispanic Black compared with non-Hispanic White, and interaction AHR, 0.55 [95% CI, 0.34-0.89] for Hispanic compared with non-Hispanic White). Similar patterns were observed for MHD- and SUD-related PPR, although rural interactions were not statistically significant.

**Conclusions and Relevance:**

In this cohort study of individuals giving birth in South Carolina, racial and ethnic disparities associated with PPR were pronounced among urban residents and attenuated in rural areas, suggesting that geographic context may modify these disparities.

## Key Points

**Question:**

Do individual residential rurality and individual race and ethnicity intersect with the risk of postpartum readmission (PPR) within 1 year post partum?

**Findings:**

In this cohort study of 190 645 births in South Carolina from January 2018 to December 31, 2021, compared with non-Hispanic White individuals, non-Hispanic Black individuals in urban areas had higher risk of all-cause PPR, whereas Hispanic individuals had lower risk. Rural residence was associated with higher all-cause PPR risk but also with attenuated racial and ethnic disparities.

**Meaning:**

Findings suggest that Black individuals in urban areas face the highest risk of all-cause PPR, rural living is associated with increased readmission risk overall, and, while rurality may be associated with decreases in some racial disparities, significant inequities remain.

## Teaser

This cohort study assesses whether individual residential rurality at time of delivery and individual race and ethnicity are associated with the risk of all-cause, mental health disorder–related, and substance use disorder–related postpartum readmission within 1 year of delivery.

## Introduction

Many individuals encounter challenges in accessing mental health and substance use treatment after childbirth, with these challenges being especially severe in rural communities.^1^ People in these populations face limited access to health care facilities^2^ and limited number of health care facilities specializing in obstetric care.^3^ Compounding these barriers are the social stigma surrounding mental health disorders (MHDs) and substance use disorders (SUDs) that may discourage people affected by these conditions from seeking treatment, further exacerbating disparities in care.^4^ According to data from maternal mortality review committees across 38 states in 2020, 23% of maternal deaths were due to mental health conditions,^5^ underscoring the urgent need for addressing the impact of mental health in perinatal outcomes.

Given these gaps in care, measuring postpartum readmission (PPR) can serve as a key but complex indicator for health care system effectiveness. What makes this indicator complex is that PPR can reflect appropriate access to care, unmet needs, or poor continuity of care. The postpartum period is an essential time for individual recovery and sustained long-term well-being. Individuals are especially vulnerable during this time, with racial and ethnic disparities in adverse outcomes becoming more evident.^6^ After childbirth, 2% of individuals throughout the US experience PPR.^7^ Black individuals were reported to have as much as 80% higher risk of PPR compared with White individuals in a nationwide study of 11 million births from 2012 through 2014.^8^ Prior research has identified important associations between perinatal behavioral health conditions and PPR, but few studies have explored how these outcomes vary at the intersection of rurality and race and ethnicity. For example, one study examined racial and geographic disparities specific to MHD- and SUD- related PPR in South Carolina, finding increased risk among rural and Black individuals, with racial and geographic disparities in risk, but did not assess all-cause readmission or test interaction effects.^9^ Similarly, another study used national data to show that illicit substance use during pregnancy was associated with increased PPR but did not examine how these risks vary by rurality or racial and ethnic identity.^10^

The present study seeks to fill that gap by examining how residential rurality and racial and ethnic identity intersect to impact all-cause, MHD-, and SUD-related PPR within the first year post partum among individuals in South Carolina. This state ranks eighth highest in the nation for maternal mortality^11^ and provides a valuable setting to examine this question due to its large rural population and persistent racial disparities in individual health outcomes. Moreover, from 2018 to 2021, 12.6% of pregnancy-related deaths in South Carolina were attributed to MHD or SUD.^12^

## Methods

### Data Sources

Using vital records linked to all-payer hospital data, we conducted a statewide retrospective cohort study of individuals giving birth in South Carolina. Deidentified data with unique identifiers were provided by the South Carolina Office of Revenue and Fiscal Affairs. We merged inpatient records from all-payer claims data with birth certificate registries, using unique patient identifiers and childbirth dates. All-payer hospital data include inpatient records for individuals covered by both public (eg, Medicaid, Medicare) and private insurers, allowing for a comprehensive assessment of health outcomes across diverse socioeconomic and insurance groups. This approach enhances generalizability and minimizes selection bias related to insurance coverage. Childbirth hospitalizations were identified using *International Classification of Disease, Tenth Revision, Clinical Modification (ICD-10-CM)* diagnoses and procedure codes, diagnosis related group codes, and *Current Procedural Terminology* codes for delivery-related diagnosis and procedures using previously published algorithms.^13^ The study followed the Strengthening the Reporting of Observational Studies in Epidemiology (STROBE) reporting guideline for cohort studies and was completed August 17, 2025. This study was deemed exempt from review and the requirement for obtaining informed consent was waived by the University of South Carolina, Columbia, Institutional Review Board, as the study involved secondary analysis of deidentified data.

### Study Population

The study population included 203 682 distinct individuals with 248 456 unique childbirth hospitalization records (eFigure in Supplement 1). First, we included unique individuals with at least 1 childbirth record having births 160 or more days apart. We then included unique individuals with a childbirth between January 1, 2018, and December 31, 2021. Unique individuals who had a birth in December 2021 but were discharged in January 2022 were excluded (n = 181) to allow for 1 full year of postpartum follow-up. We combined the remaining records with birth certificate data, excluding unlinkable data or nonhospital births (1.2%). We also excluded records with missing or unknown data on the following covariates: race and ethnicity (0.7%), Kotelchuck Index (1.0%), and educational attainment (0.3%). After deleting records with missing individual characteristics, our process resulted in 190 645 unique births to 166 330 individuals. To capture 1 full year of follow-up, postpartum records for each birth were included through December 31, 2022.

### Outcomes and Measures

Our primary outcomes of interest were the rates and timing of 3 PPR outcomes, all-cause, MHD-related, and SUD-related, within 42, 90, 180, and 365 days post partum. We defined all-cause PPR as inpatient records with the admission date occurring after the initial childbirth discharge date based on *ICD* codes identified in previous methods^7^ and converted those codes to *ICD-10-CM* codes as was done in a previous study conducted by a member of our team (C.M.T.) (eTable 1 in Supplement 1).^14^ We identified MHD- and SUD-related PPRs based on *ICD-10-CM* codes published in a prior PPR study (eTable 2 in Supplement 1)^9^ and included admissions in which these codes appeared in any diagnosis field. MHD PPRs included admissions due to mental health and behavioral disorders caused by psychoactive substance use, other SUDs, psychosis, mood disorders, anxiety- and stress-related disorders, sleep disorders, and other mental disorders during the postpartum period. SUD-related PPR included admissions due to opioids, cocaine, stimulants, and other SUDs. In the event that there was more than 1 readmission event, for each outcome only the first readmission event during the follow-up period was counted. Follow-up time was defined as the number of days from the initial childbirth discharge to the first qualifying readmission event or censoring at 365 days post partum, whichever occurred first.

We examined individual race and ethnicity and residential location as our primary exposures of interest. Individual race and ethnicity data were self-reported and derived from the birth certificate. Race and ethnicity included Hispanic, non-Hispanic Black (Black), non-Hispanic White (White), and non-Hispanic other (other), which included American Indian, Alaska Native, Asian, Native Hawaiian, and Other Pacific Islander. Individual residential location (rural vs urban) was measured based on the county of residence at the time of initial childbirth hospital stay using 2023 Rural-Urban Continuum Codes.

We selected covariates based on their established associations with PPR in prior research.^7,14,15^ These covariates included individual age, insurance type, mode of delivery, the Adequacy of Prenatal Care Utilization Index (commonly known as the Kotelchuck Index), parity, childbirth initial hospitalization length of stay, educational attainment, year of birth, and obstetric comorbidity index. The Kotelchuck Index assesses both the timing of initiation of prenatal care and the number of visits received, adjusted for gestational age: inadequate care indicates less than 50% of expected visits, intermediate care indicates 50% to 79% of expected visits, adequate care indicates 80% to 109% of expected visits, and adequate plus care indicates 110% or more of expected visits.^16^ The obstetric comorbidity index, categorized in our study as 0, 1 to 8, 9 to 14, and 15 or more, is a scoring system developed to quantify the magnitude and to predict severe maternal morbidity^17^; in this study, it was used to adjust for underlying individual health complexity. We treated the index as a categorical variable because previous research has shown that its association with adverse individual outcomes is not linear. Categorization allows for better modeling of potential threshold effects, in which risk may disproportionately increase at higher comorbidity levels, and more accurately reflects the nonlinear association between comorbidity burden and PPR risk. Prior research has also shown that PPR patterns and associated disparities can shift over time, reinforcing the importance of controlling for birth year in longitudinal analyses.^14^ Year of birth was included as a categorical covariate to adjust for fixed events associated with calendar year, including the onset and early phases of the COVID-19 pandemic.

### Statistical Analysis

Demographic characteristics by individual race and ethnicity and individual residential rurality (rural vs urban) were summarized using descriptive statistics. Differences in continuous variables were assessed using analysis of variance, and differences in categorical variables were examined using Pearson χ^2^ tests. We compared the distributions of individual characteristics between individuals with or without PPR outcomes using χ^2^ tests. Kaplan-Meier curves were generated to estimate the probability of remaining free from PPR for the 1-year postpartum period, stratified by race or ethnicity and individual residential rurality. Group differences in probability distributions were assessed using 2-sided log-rank tests.

Cox proportional hazards models were used to assess associations between individual characteristics and time to PPR outcomes. Time to event was defined as the number of days from the initial birth hospitalization discharge to the first PPR or censoring at 365 days, whichever occurred first. Kaplan-Meier curves display the cumulative incidence of readmission over time, which increases as more events occur across the follow-up period. Hazard ratios (HRs) and 95% CIs were estimated for each characteristic. Proportional hazards assumptions were evaluated using Schoenfeld residuals, and *P* values were derived using the Wald test.

We separately analyzed each of 3 PPR outcomes: all-cause, MHD-related, and SUD-related. We first estimated crude HRs for each individual characteristic using univariable Cox proportional hazards models. Next, we fit an unadjusted multivariable model that included individual residence (rural vs urban), race and ethnicity, and their interaction. Finally, we fit fully adjusted models including individual race and ethnicity, residence, the race-by-residence interaction term, and the covariates insurance type, mode of delivery, Kotelchuck Index, parity, length of stay for the birth hospitalization, educational attainment, year of birth, and obstetric comorbidity index. In these models, estimated race and ethnicity represented associations among individuals living in urban residents, while the interaction terms indicated how those associations differed among individuals living in rural areas. Statistical significance was defined as a 2-sided *P* < .05. All analyses were conducted using R, version 4.3.2 (R Project for Statistical Computing). Descriptive statistics and *P* values were calculated using the gtsummary package (version 2.0.4). Kaplan-Meier curves and Cox proportional hazards models were performed using the survival package (version 3.7.0) and visualized with survminer (version 0.4.9).

## Results

### Study Population

Our study cohort consisted of 190 645 births to 166 330 unique individuals (mean [SD] age, 28.2 [5.8] years) in South Carolina from January 1, 2018, to December 31, 2021, of whom 30.9% were Black, 4.9% were Hispanic, 57.1% were White, and 7.1% were other race and ethnicity (Table 1). The highest percentages of individuals in our cohort were between 25 and 29 years of age (30.4%), had some college education (32.9%), had a primary payer at birth of Medicaid (46.3%), and had an initial birth hospitalization stay of 2 to 3 days (76.4%). In total, 27 961 births to individuals residing in rural areas accounted for 14.7% of our cohort, with 162 684 (85.3%) births to individuals residing in urban areas (Table 2). All-cause PPR occurred in 1.8% of individuals within 42 days after initial birth hospitalization discharge, 2.2% of individuals within 90 days, 2.6% of individuals within 180 days, and 4.7% of individuals up to 1 year (Table 3). Among births to individuals residing in rural residential areas, 5.5% had an all-cause PPR up to 365 days post partum. In addition, 1.5% had MHD-related PPR and 0.8% had SUD-related PPR. eTables 3-5 in Supplement 1 present the descriptive statistics on MHD- and SUD-related PPR and all 3 outcomes combined, respectively, up to 1 year after initial birth hospitalization discharge.

**Table 1.** Characteristics of Birthing Individuals, Overall and by Race and Ethnicity in South Carolina, 2018-2021

Individual characteristic	Births, No. (%)^a^^,^^b^				
	Overall (N = 190 645)	Non-Hispanic Black (n = 58 859)	Non-Hispanic White (n = 108 812)	Hispanic (n = 9400)	Other race and ethnicity^c^ (n = 13 574)
Residence rurality					
Rural	27 961 (14.7)	11 937 (20.3)	13 991 (12.9)	722 (7.7)	1311 (9.7)
Urban	162 684 (85.3)	46 922 (79.7)	94 821 (87.1)	8678 (92.3)	12 263 (90.3)
Age, y					
<20	11 655 (6.1)	4953 (8.4)	5008 (4.6)	836 (8.9)	858 (6.3)
20-24	42 914 (22.5)	16 278 (27.7)	21 708 (20.0)	2294 (24.4)	2634 (19.4)
25-29	57 921 (30.4)	18 219 (31.0)	33 392 (30.7)	2545 (27.1)	3765 (27.7)
30-34	49 997 (26.2)	12 383 (21.0)	31 747 (29.2)	2123 (22.6)	3744 (27.6)
≥35	28 158 (14.8)	7026 (11.9)	16 957 (15.6)	1602 (17.0)	2573 (19.0)
Educational attainment					
Bachelor’s degree	34 976 (18.3)	5403 (9.2)	26 450 (24.3)	638 (6.8)	2485 (18.3)
Graduate school	19 707 (10.3)	2821 (4.8)	14 989 (13.8)	277 (2.9)	1620 (11.9)
High school diploma	49 102 (25.8)	20 984 (35.7)	22 012 (20.2)	2837 (30.2)	3269 (24.1)
No high school diploma	24 072 (12.6)	7212 (12.3)	10 135 (9.3)	3926 (41.8)	2799 (20.6)
Some college	62 788 (32.9)	22 439 (38.1)	35 226 (32.4)	1722 (18.3)	3401 (25.1)
Primary payer at birth					
Private	76 651 (40.2)	15 725 (26.7)	55 115 (50.7)	1306 (13.9)	4505 (33.2)
Medicaid	88 240 (46.3)	37 264 (63.3)	37 330 (34.3)	6954 (74.0)	6692 (49.3)
Other public	21 998 (11.5)	5409 (9.2)	14 237 (13.1)	641 (6.8)	1711 (12.6)
Uninsured	3756 (2.0)	461 (0.8)	2130 (2.0)	499 (5.3)	666 (4.9)
Cesarean delivery	63 389 (33.2)	20 990 (35.7)	35 465 (32.6)	2696 (28.7)	4238 (31.2)
Trimester prenatal care began					
First	144 710 (75.9)	42 076 (71.5)	87 795 (80.7)	5817 (61.9)	9022 (66.5)
Second	35 407 (18.6)	13 053 (22.2)	16 369 (15.0)	2619 (27.9)	3366 (24.8)
Third	10 528 (5.5)	3730 (6.3)	4648 (4.3)	964 (10.3)	1186 (8.7)
Kotelchuck Index					
Inadequate	9995 (5.2)	3436 (5.8)	5045 (4.6)	598 (6.4)	916 (6.7)
Intermediate	32 128 (16.9)	11 839 (20.1)	14 123 (13.0)	2768 (29.4)	3398 (25.0)
Adequate	53 864 (28.3)	14 032 (23.8)	33 344 (30.6)	2713 (28.9)	3775 (27.8)
Adequate Plus	94 658 (49.7)	29 552 (50.2)	56 300 (51.7)	3321 (35.3)	5485 (40.4)
Parity					
Primary birth	75 209 (39.4)	21 685 (36.8)	45 156 (41.5)	2917 (31.0)	5451 (40.2)
1 Previous live birth	60 434 (31.7)	16 984 (28.9)	36 428 (33.5)	2815 (29.9)	4207 (31.0)
≥2 Previous live births	55 002 (28.9)	20 190 (34.3)	27 228 (25.0)	3668 (39.0)	3916 (28.8)
Childbirth hospitalization length of stay, d					
0-1	19 261 (10.1)	4037 (6.9)	12 920 (11.9)	746 (7.9)	1558 (11.5)
2-3	145 666 (76.4)	45 098 (76.6)	82 775 (76.1)	7439 (79.1)	10 354 (76.3)
≥4	25 718 (13.5)	9724 (16.5)	13 117 (12.1)	1215 (12.9)	1662 (12.2)
Year of birth					
2018	47 607 (25.0)	14 867 (25.3)	27 232 (25.0)	2158 (23.0)	3350 (24.7)
2019	48 714 (25.6)	15 200 (25.8)	27 726 (25.5)	2303 (24.5)	3485 (25.7)
2020	47 684 (25.0)	14 946 (25.4)	26 785 (24.6)	2421 (25.8)	3532 (26.0)
2021	46 640 (24.5)	13 846 (23.5)	27 069 (24.9)	2518 (26.8)	3207 (23.6)
Obstetric comorbidity index score^d^					
0	70 484 (37.0)	17 804 (30.2)	42 838 (39.4)	3919 (41.7)	5923 (43.6)
1-8	59 837 (31.4)	16 523 (28.1)	36 629 (33.7)	2756 (29.3)	3929 (28.9)
9-14	40 508 (21.2)	15 870 (27.0)	19 894 (18.3)	2034 (21.6)	2710 (20.0)
≥15	19 816 (10.4)	8662 (14.7)	9451 (8.7)	691 (7.4)	1012 (7.5)

^a^ All characteristics were significantly different across race and ethnicity groups at *P* < .001 as calculated using Pearson χ^2^ tests.

^b^ Column percentages presented.

^c^ Other race and ethnicity groups included American Indian or Alaska Native, Asian, Native Hawaiian, Other Pacific Islander, and multiple races.

^d^ Higher scores indicate greater maternal health risk due to more severe preexisting or pregnancy-related conditions.

**Table 2.** Characteristics of Birthing Individuals, Overall and by Residence Location in South Carolina, 2018-2021

Individual characteristic	Births, No. (%)^a^^,^^b^			*P* value
	Overall (N = 190 645	Rural (n = 27 961)	Urban (n = 162 684)	
Race or ethnicity				
Non-Hispanic Black	58 859 (30.9)	11 937 (42.7)	46 922 (28.8)	<.001
Non-Hispanic White	108 812 (57.1)	13 991 (50.0)	94 821 (58.3)	
Hispanic	9400 (4.9)	722 (2.6)	8678 (5.3)	
Other^c^	13 574 (7.1)	1311 (4.7)	12 263 (7.5)	
Individual age, y				
<20	11 655 (6.1)	2402 (8.6)	9253 (5.7)	<.001
20-24	42 914 (22.5)	8104 (29.0)	34 810 (21.4)	
25-29	57 921 (30.4)	8674 (31.0)	49 247 (30.3)	
30-34	49 997 (26.2)	5745 (20.5)	44 252 (27.2)	
≥35	28 158 (14.8)	3036 (10.9)	25 122 (15.4)	
Educational attainment				
Bachelor’s degree	34 976 (18.3)	2982 (10.7)	31 994 (19.7)	<.001
Graduate school	19 707 (10.3)	1440 (5.2)	18 267 (11.2)	
High school diploma	49 102 (25.8)	9487 (33.9)	39 615 (24.4)	
No high school diploma	24 072 (12.6)	4190 (15.0)	19 882 (12.2)	
Some college	62 788 (32.9)	9862 (35.3)	52 926 (32.5)	
Primary payer at birth				
Private	76 651 (40.2)	9222 (33.0)	67 429 (41.4)	<.001
Medicaid	88 240 (46.3)	15 337 (54.9)	72 903 (44.8)	
Other public	21 998 (11.5)	2880 (10.3)	19 118 (11.8)	
Uninsured	3756 (2.0)	522 (1.9)	3234 (2.0)	
Cesarean delivery	63 389 (33.2)	9438 (33.8)	53 951 (33.2)	.053
Trimester prenatal care began				
First	144 710 (75.9)	20 838 (74.5)	123 872 (76.1)	<.001
Second	35 407 (18.6)	5443 (19.5)	29 964 (18.4)	
Third	10 528 (5.5)	1680 (6.0)	8848 (5.4)	
Kotelchuck Index				
Inadequate	9995 (5.2)	1384 (4.9)	8611 (5.3)	<.001
Intermediate	32 128 (16.9)	5114 (18.3)	27 014 (16.6)	
Adequate	53 864 (28.3)	6874 (24.6)	46 990 (28.9)	
Adequate Plus	94 658 (49.7)	14 589 (52.2)	80 069 (49.2)	
Parity				
Primary birth	75 209 (39.4)	10 647 (38.1)	64 562 (39.7)	<.001
1 Previous live birth	60 434 (31.7)	8623 (30.8)	51 811 (31.8)	
≥2 Previous live births	55 002 (28.9)	8691 (31.1)	46 311 (28.5)	
Childbirth hospitalization length of stay, d				
0-1	19 261 (10.1)	2707 (9.7)	16 554 (10.2)	<.001
2-3	145 666 (76.4)	21 966 (78.6)	123 700 (76.0)	
≥4	25 718 (13.5)	3288 (11.8)	22 430 (13.8)	
Year of birth				
2018	47 607 (25.0)	7112 (25.4)	40 495 (24.9)	.03
2019	48 714 (25.6)	7200 (25.8)	41 514 (25.5)	
2020	47 684 (25.0)	6995 (25.0)	40 689 (25.0)	
2021	46 640 (24.5)	6654 (23.8)	39 986 (24.6)	
Obstetric comorbidity index score^d^				
0	70 484 (37.0)	10 287 (36.8)	60 197 (37.0)	.051
1-8	59 837 (31.4)	8954 (32.0)	50 883 (31.3)	
9-14	40 508 (21.2)	5813 (20.8)	34 695 (21.3)	
≥15	19 816 (10.4)	2907 (10.4)	16 909 (10.4)	

^a^ Column percentages presented.

^b^ *P* values were calculated to compare individual characteristics between rural and urban birthing individuals using Pearson χ^2^ tests.

^c^ Other race and ethnicity groups included American Indian or Alaska Native, Asian, Native Hawaiian, Other Pacific Islander, and multiple races.

^d^ Higher scores indicate greater maternal health risk due to more severe preexisting or pregnancy-related conditions.

**Table 3.** Percentages of All-Cause PPR, by Individual Characteristic at 42, 90, 180, and 365 Days Post Partum in South Carolina, 2018-2021

Individual characteristic	Births, No. (%)^a^^,^^b^								
	Overall (N = 190 645)	42 d		90 d		180 d		365 d	
		n = 3500	*P* value	n = 4155	*P* value	n = 5038	*P* value	n = 8976	*P* value
Race or ethnicity									
Hispanic	9400 (4.9)	125 (1.3)	<.001	167 (1.8)	<.001	206 (2.2)	<.001	363 (3.9)	<.001
Non-Hispanic Black	58 859 (30.9)	1579 (2.7)		1819 (3.1)		2154 (3.7)		3918 (6.7)	
Non-Hispanic White	108 812 (57.1)	1628 (1.5)		1951 (1.8)		2404 (2.2)		4218 (3.9)	
Other^c^	13 574 (7.1)	168 (1.2)		218 (1.6)		274 (2.0)		477 (3.5)	
Residence rurality									
Rural	27 961 (14.7)	518 (1.9)	.83	643 (2.3)	.14	810 (2.9)	.004	1538 (5.5)	<.001
Urban	162 684 (85.3)	2982 (1.8)		3512 (2.2)		4228 (2.6)		7438 (4.6)	
Individual age, y									
<20	11 655 (6.1)	167 (1.4)	<.001	232 (2.0)	<.001	312 (2.7)	<.001	777 (6.7)	<.001
20-24	42 914 (22.5)	696 (1.6)		886 (2.1)		1136 (2.6)		2474 (5.8)	
25-29	57 921 (30.4)	964 (1.7)		1141 (2.0)		1408 (2.4)		2514 (4.3)	
30-34	57 921 (30.4)	948 (1.9)		1087 (2.2)		1262 (2.5)		1997 (4.0)	
≥35	57 921 (30.4)	725 (2.6)		809 (2.9)		920 (3.3)		1214 (4.3)	
Educational attainment									
Bachelor’s degree	34 976 (18.3)	511 (1.5)	<.001	566 (1.6)	<.001	656 (1.9)	<.001	924 (2.6)	<.001
Graduate school	19 707 (10.3)	297 (1.5)		332 (1.7)		375 (1.9)		491 (2.5)	
High school diploma	49 102 (25.8)	958 (2.0)		1171 (2.4)		1431 (2.9)		2859 (5.8)	
No high school diploma	24 072 (12.6)	421 (1.7)		545 (2.3)		729 (3.0)		1599 (6.6)	
Some college	62 788 (32.9)	1313 (2.1)		1541 (2.5)		1847 (2.9)		3103 (4.9)	
Primary payer at birth									
Private	76 651 (40.2)	1265 (1.7)	<.001	1453 (1.9)	<.001	1685 (2.2)	<.001	2545 (3.3)	<.001
Medicaid	88 240 (46.3)	1806 (2.0)		2197 (2.5)		2728 (3.1)		5385 (6.1)	
Other public	21 998 (11.5)	392 (1.8)		463 (2.1)		570 (2.6)		922 (4.2)	
Uninsured	3756 (2.0)	37 (1.0)		42 (1.1)		55 (1.5)		124 (3.3)	
Cesarean delivery	63 389 (33.2)	1727 (2.7)	<.001	1975 (3.1)	<.001	2319 (3.7)	<.001	3520 (5.6)	<.001
Trimester prenatal care began									
First	144 710 (75.9)	2678 (1.9)	.61	3141 (2.2)	.49	3778 (2.6)	.29	6572 (4.5)	<.001
Second	35 407 (18.6)	640 (1.8)		796 (2.2)		976 (2.8)		1850 (5.2)	
Third	10 528 (5.5)	182 (1.7)		218 (2.1)		284 (2.7)		554 (5.3)	
Kotelchuck Index									
Inadequate	9995 (5.2)	166 (1.7)	<.001	200 (2.0)	<.001	257 (2.6)	<.001	498 (5.0)	<.001
Intermediate	32 128 (16.9)	583 (1.8)		716 (2.2)		907 (2.8)		1827 (5.7)	
Adequate	53 864 (28.3)	742 (1.4)		891 (1.7)		1084 (2.0)		1963 (3.6)	
Adequate Plus	94 658 (49.7)	2009 (2.1)		2348 (2.5)		2790 (2.9)		4688 (5.0)	
Parity									
Primary birth	75 209 (39.4)	1426 (1.9)	<.001	1677 (2.2)	<.001	2006 (2.7)	<.001	3571 (4.7)	<.001
1 Previous live birth	60 434 (31.7)	952 (1.6)		1156 (1.9)		1426 (2.4)		2535 (4.2)	
≥2 Previous live births	55 002 (28.9)	1122 (2.0)		1322 (2.4)		1606 (2.9)		2870 (5.2)	
Childbirth hospitalization length of stay, d									
0-1	19 261 (10.1)	291 (1.5)	<.001	350 (1.8)	<.001	426 (2.2)	<.001	786 (4.1)	<.001
2-3	145 666 (76.4)	2253 (1.5)		2714 (1.9)		3336 (2.3)		6239 (4.3)	
≥4	25 718 (13.5)	956 (3.7)		1091 (4.2)		1276 (5.0)		1951 (7.6)	
Year of birth									
2018	47 607 (25.0)	880 (1.8)	.02	1057 (2.2)	.02	1313 (2.8)	.007	2362 (5.0)	.002
2019	48 714 (25.6)	885 (1.8)		1047 (2.1)		1265 (2.6)		2222 (4.6)	
2020	47 684 (25.0)	814 (1.7)		970 (2.0)		1171 (2.5)		2149 (4.5)	
2021	46 640 (24.5)	921 (2.0)		1081 (2.3)		1289 (2.8)		2243 (4.8)	
Obstetric comorbidity index score^d^									
0	70 484 (37.0)	658 (0.9)	<.001	823 (1.2)	<.001	1046 (1.5)	<.001	2181 (3.1)	<.001
1-8	59 837 (31.4)	1116 (1.9)		1335 (2.2)		1598 (2.7)		2752 (4.6)	
9-14	40 508 (21.2)	892 (2.2)		1053 (2.6)		1248 (3.1)		2237 (5.5)	
≥15	19 816 (10.4)	834 (4.2)		944 (4.8)		1146 (5.8)		1806 (9.1)	

Abbreviation: PPR, postpartum readmission.

^a^ *P* values were calculated to compare individual characteristics with all-cause PPR up to 365 days post partum using Pearson χ^2^ tests.

^b^ Row percentages presented.

^c^ Other race and ethnicity groups included American Indian or Alaska Native, Asian, Native Hawaiian, Other Pacific Islander, and multiple races.

^d^ Higher scores indicate greater maternal health risk due to more severe preexisting or pregnancy-related conditions.

### All-Cause Postpartum Hospital Readmission

Compared with White individuals, in our crude models (Table 4; eTable 6 in Supplement 1), Black individuals had significantly higher hazard of all-cause PPR (crude HR, 1.74 [95% CI, 1.67-1.82]), whereas Hispanic individuals had a similar hazard compared with White individuals (crude HR, 1.00 [95% CI, 0.89-1.11]). Rural residence was also associated with a higher hazard of all-cause readmission (crude HR, 1.21 [95% CI, 1.14-1.27]).

**Table 4.** Hazard Ratios of All Cause, Mental Health Disorder–, and Substance Use Disorder–Related PPR, by Individual Race, Ethnicity, and Residence Location in South Carolina, 2018-2021

Individual characteristic	Hazard ratio (95% CI)					
	All-cause		Mental health disorder		Substance use disorder	
	Crude^a^	Adjusted, urban^b^	Crude^a^	Adjusted, urban^b^	Crude^a^	Adjusted, urban^b^
Race or ethnicity^c^						
Non-Hispanic Black	1.74 (1.67-1.82)	1.38 (1.31-1.45)	1.08 (1.00-1.17)	0.74 (0.68-0.82)	1.11 (1.00-1.23)	0.67 (0.60-0.76)
Hispanic	1.00 (0.89-1.11)	0.83 (0.74-0.93)	0.36 (0.28-0.47)	0.24 (0.18-0.32)	0.23 (0.14-0.36)	0.12 (0.07-0.18)
Other^d^	0.90 (0.82-1.00)	0.84 (0.76-0.93)	0.539 (0.45-0.65)	0.454 (0.37-0.56)	0.43 (0.32-0.56)	0.30 (0.22-0.41)
Residence rurality^c^						
Rural	1.21 (1.14-1.27)	1.15 (1.06-1.25)	1.17 (1.06-1.29)	1.06 (0.93-1.20)	1.40 (1.24-1.59)	1.14 (0.97-1.35)
Interaction term						
Non-Hispanic Black × rural^e^	NA	0.86 (0.77-0.97)	NA	0.92 (0.75-1.13)	NA	0.99 (0.76-1.27)
Hispanic × rural^f^	NA	0.55 (0.34-0.89)	NA	0.47 (0.11-1.92)	NA	0.62 (0.08-4.69)
Other race and ethnicity × rural^g^	NA	1.04 (0.78-1.38)	NA	1.04 (0.58-1.84)	NA	1.24 (0.57-2.68)

Abbreviations: NA, not applicable; PPR, postpartum readmission.

^a^ Crude models fitted respectively without accounting for other confounders.

^b^ Covariates being controlled included insurance type, mode of delivery, Kotelchuck Index, parity, initial hospitalization length of stay, educational attainment, year of birth, and obstetric comorbidity index.

^c^ Reference groups: Non-Hispanic White for race and ethnicity; urban for residence rurality.

^d^ Other race and ethnicity groups included American Indian or Alaska Native, Asian, Native Hawaiian, Other Pacific Islander, and multiple races.

^e^ *P* values for interaction terms for all-cause PPR are .01 for Black, .02 for Hispanic, and .80 for other race and ethnicity.

^f^ *P* values for interaction terms for mental health disorder–related PPR are .43 for Black, .29 for Hispanic, and .90 for other race and ethnicity.

^g^ *P* values for interaction terms for substance use disorder–related PPR are .91 for Black, .64 for Hispanic, and .59 for other race and ethnicity.

After adjusting for individual characteristics and including interaction terms, Black individuals had higher risk of all-cause PPR compared with White individuals in urban areas (adjusted HR [AHR], 1.38 [95% CI, 1.31-1.45]), whereas Hispanic individuals had lower risk (AHR, 0.83 [95% CI, 0.74-0.93]) (Table 4; eTable 6 in Supplement 1). Rural residence was independently associated with higher risk (AHR, 1.15 [95% CI, 1.06-1.25]). However, living in rural areas was associated with reduced racial and ethnic disparities in all-cause PPR: the Black compared with White disparity was attenuated (interaction AHR, 0.86 [95% CI, 0.77-0.97), as was the Hispanic compared with White disparity (interaction AHR, 0.55 [95% CI, 0.34-0.89]).

### MHD- and SUD-Related Postpartum Hospital Readmissions

Up to 1 year post partum, MHD- and SUD-related PPR varied significantly by race and ethnicity. In crude models (Table 4; eTable 6 in Supplement 1), compared with White individuals, Black individuals had nonsignificantly higher hazard of MHD-related PPR (HR, 1.08 [95% CI, 1.00-1.17]) and SUD-related PPR (HR, 1.11 [95% CI, 1.00-1.23]). Hispanic individuals had reduced hazards of both MHD-related PPR (HR, 0.36 [95% CI, 0.28-0.47]) and SUD-related PPR (HR, 0.23 [95% CI, 0.14-0.36]).

After adjusting for individual characteristics and including interaction terms, among urban residents (the reference group), Black individuals had 25.5% lower hazard of MHD-related PPR (AHR, 0.74 [95% CI, 0.68-0.82]) and 32.7% lower hazard of SUD-related PPR (AHR, 0.67 [95% CI, 0.60-0.76]) compared with White individuals (Table 4; eTable 6 in Supplement 1). Hispanic individuals also had significantly reduced hazards of MHD-related PPR (AHR, 0.24 [95% CI, 0.18-0.32]) and SUD-related PPR (AHR, 0.12 [95% CI, 0.07-0.18]). A proportion of individuals experienced more than 1 PPR during the follow-up period, which is detailed by outcome category in eTable 7 in Supplement 1.

The racial and ethnic disparities appeared attenuated in rural areas, although interactions were not statistically significant. For example, the Black compared with White disparity in MHD-PPR was reduced in rural areas (interaction AHR, 0.92 [95% CI, 0.75-1.13]), as was the SUD-PPR disparity (interaction AHR, 0.99 [95% CI, 0.76-1.27]). Similarly, Hispanic compared with White disparities in MHD-PPR (interaction AHR, 0.47 [95% CI, 0.11-1.92]) and SUD-PPR (interaction AHR, 0.62 [95% CI, 0.08-4.69]) were also attenuated in rural settings and were not statistically significant.

Kaplan-Meier curves for all-cause, MHD-related, and SUD-related PPRs up to 1 year post partum revealed clear racial and ethnic disparities in urban areas, with Black individuals experiencing the lowest probabilities of remaining free of PPR across all outcomes (Figure). In contrast, these disparities were attenuated among rural residents, particularly for all-cause readmissions. Hispanic individuals consistently had the highest probabilities of remaining free of PPR across both settings and outcomes.

**Figure.**
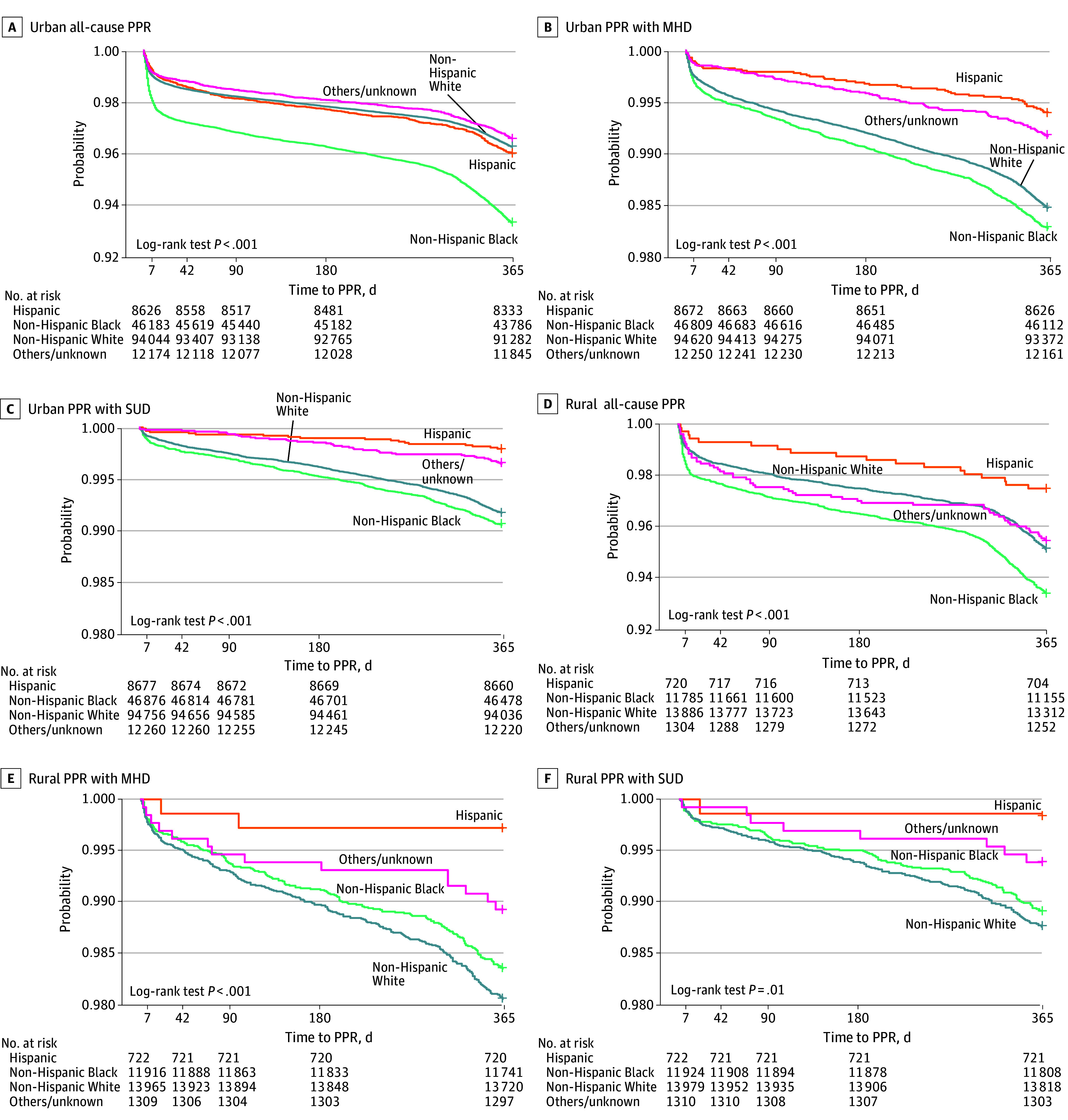
Kaplan-Meier Curves Showing Probability of All-Cause, Mental Health Disorder (MHD)–Related, and Substance Use Disorder (SUD)–Related Postpartum Readmission (PPR), Stratified by Individual Residence Location, Race, and Ethnicity Curves display the probability of remaining free from postpartum readmission for 1 year following childbirth, stratified by race and ethnicity and residence (urban vs rural). Log-rank test *P value*s indicate statistically significant differences in probability distributions across groups. Race and ethnicity categories are mutually exclusive. Residence was classified based on individual county of residence at the time of delivery. Higher curves indicate lower risk of readmission over time.

## Discussion

Our statewide, population-based cohort study using linked vital records and hospital discharge data found that all-cause PPR increased during a 1-year follow-up, as expected in time-to-event analyses, reaching 4.8% by 1 year post partum. This pattern reflects the accumulation of first readmission events over time, rather than changes in the rate of readmission at specific intervals. To better understand the factors associated with PPR risk, we examined how these patterns vary across racial and ethnic groups and how residential location may interact with these disparities.

### Racial and Ethnic Disparities in PPR

The HR for all-cause PPR was greatest for Black individuals even after adjustment, indicating persistent disparities. In contrast, disparities in MHD- and SUD-related PPR by race and ethnicity were attenuated after adjustment. Both Black and Hispanic individuals had decreased hazards of MHD- and SUD-related PPR after adjustment, suggesting that observed differences in unadjusted models may be largely explained by the other individual and clinical characteristics. Our finding that Hispanic individuals consistently had lower hazards of both MHD- and SUD-related PPR in both crude and adjusted models suggests that there may be unmeasured protective factors (eg, differences in health care–seeking behaviors, strong social support, and the Hispanic paradox^18^) associated with reduced readmissions for these outcomes compared with White individuals. The data suggest that racial and ethnic disparities in PPR exist in urban areas, with some evidence that these disparities are attenuated in rural settings based on interaction terms. However, further research is needed to determine whether disparities are truly more prevalent in urban South Carolina.

### Rural-Urban Disparities in PPR

Several studies have demonstrated that maternal morbidity and mortality are elevated in rural populations,^19,20^ with a large amount of this morbidity and mortality associated with individuals losing access to care.^21^ Few studies have explored the association between individual residence and PPR.^22^ Interventions such as telehealth services are increasingly proposed to address access challenges,^23,24^ particularly in rural areas, but uptake of these services remains unequal across populations. Our study found that rural residence was not independently associated with PPR risk after adjusting for individual and clinical factors. This finding suggests that individual-level characteristics, rather than geography alone, may be the primary factor associated with PPR disparities in our sample. Rurality modified some associations; however, these modifications were not uniform across the sample. Future work should continue to investigate how structural and geographic factors intersect with individual risk to inform targeted postpartum care strategies.

These findings from South Carolina suggest that existing strategies targeting rural populations may be mitigating the risks associated with all-cause PPR. These results emphasize the increased need for interventions that address issues such as access to care and MHD and SUD treatment, especially in non-Hispanic White populations, and improving community support. A recent scoping review highlighted several interventions targeting perinatal care in rural communities, ranging from increased educational attainment and health care frequency to social support networks.^25^ Future research should investigate how these existing interventions may function as protective mechanisms against PPR and how they can be effectively scaled across diverse geographic settings. Additionally, further work is needed to understand how factors such as health care access, health care professional availability, and the delivery of culturally responsive care contribute to the persistence of disparities in postpartum outcomes.

### Strengths and Limitations

Our study has several strengths. We extend current literature by measuring PPR up to 1 year post partum and disaggregating PPR into MHD and SUD indicators, both of which are key factors associated with maternal morbidity and mortality.^26,27^ Our study included a large proportion of Black individuals (30.9%), and their initially higher risk of MHD- and SUD-related PPR was reduced after adjusting for individual characteristics.

Our study has several limitations. While our study included data from a statewide racially and ethnically diverse population cohort, our findings may not be generalizable to other states across the US with different levels of health care access, systemic policies, or demographic compositions. We also were unable to account for unmeasured confounders, such as implicit biases, in the health care system, individual health behaviors, hospital factors or travel time to nearest hospital. These data could provide additional insight into the barriers different populations face. There is also the potential of underestimating or overestimating postpartum diagnoses due to the nature of *ICD* coding, which was originally intended for use in health care system billing.

Our study period included the early phase of the COVID-19 pandemic, which likely influenced PPRs through disruptions in health care delivery, mental health service availability, and individual care-seeking behavior. Although we adjusted for year of birth as a fixed covariate to account for temporal patterns, this approach may not fully capture the complex and evolving impacts of the pandemic. Future studies with finer-grained time variables or policy-linked data could better examine these dynamics. An additional limitation is that our measure of rurality was based on county of residence at the time of birth, which may not fully capture variation in geographic access to care. We did not include more granular metrics, such as travel time, health care professional density, or built environment characteristics (eg, hospital closures, broadband access, or transportation availability) that could influence postpartum health care utilization. Future research should incorporate finer geographic measures, such as census tract–level health care professional density or actual travel time to care facilities, to better understand how spatial access contributes to disparities in PPRs.

Our findings suggest that racial and ethnic disparities may exist in MHD- and SUD-related PPR. However, these differences should be interpreted with caution. Higher readmission rates may reflect limited access to outpatient care, fragmented transitions between inpatient and community services, or inequities in the availability of culturally responsive treatment. Conversely, lower rates of readmission, particularly among rural or marginalized populations, could signal unmet need or underutilization due to structural barriers, stigma, or geographic inaccessibility. Without clinical detail on care appropriateness or follow-up adherence, hospital readmission rates alone cannot fully distinguish between disparities in access vs health outcomes. Future studies should investigate care trajectories and outpatient engagement following discharge to better interpret these findings in context.

## Conclusions

The findings of this cohort study of PPR across rural and urban populations highlight the intersections of residential rurality and race and ethnicity on all-cause, MHD-related, and SUD-related PPR throughout the first year after hospital discharge. We initially found that rural residence was associated with all-cause, MHD-related, and SUD-related PPR, but these differences became insignificant after adjusting for individual characteristics and interactions, suggesting that rurality is not an independent factor associated with disparities in PPR. For Black and Hispanic individuals, rurality was associated with protection against PPR, underscoring the need for interventions in urban areas for Black and Hispanic populations and rural areas for White populations that address individual characteristics and other unmeasured factors associated with disparities.

## Notes

Supplement 1. **eTable 1.** Postpartum readmission *International Classification of Disease, Tenth Revision, Clinical Modification (ICD-10-CM)* diagnoses and procedure codes **eTable 2.** Postpartum mental health disorder and substance use disorder International Classification of Diseases (ICD), 10th Revision, Clinical Modification codes **eTable 3.** Proportion of mental health disorder (MHD) related postpartum readmissions by individual characteristics at 42 days, 90 days, 180 days, and 365 days postpartum, in South Carolina, 2018-2021 **eTable 4.** Proportion of substance use disorder (SUD) related postpartum readmissions by individual characteristics at 42 days, 90 days, 180 days, and 365 days postpartum, in South Carolina, 2018-2021 **eTable 5.** Proportion of all-cause, mental health disorder (MHD) and substance used disorder (SUD)-related postpartum readmissions by timing across individual race, ethnicity, and residence location groups, in South Carolina 2018-2021 **eTable 6.** Hazard ratios of all causes, mental health disorder (MHD)-, and substance used disorder (SUD)-related postpartum readmissions by individual characteristics in South Carolina **eTable 7.** Proportion of individuals who experienced multiple postpartum readmissions (PPR) within 1 year of birth **eFigure.** Study population flowchart

Supplement 2.
Data Sharing Statement
