# Supplementary material for: Variations in Postpartum Readmission by Individual Race, Ethnicity, and Rurality in South Carolina
Source: JAMA Netw Open. 2025 Dec 8;8(12):e2547455. doi: 10.1001/jamanetworkopen.2025.47455 (PMC12687097; doi:10.1001/jamanetworkopen.2025.47455)

Supplementary Online Content

Tucker CM, Ma Y, Zhang J, et al. Variations in postpartum readmission by individual race, ethnicity, and rurality in South Carolina. *JAMA Netw Open*. 2025;8(12):e2547455. doi:10.1001/jamanetworkopen.2025.47455

eTable 1. Postpartum readmission *International Classification of Disease, Tenth Revision, Clinical Modification (ICD-10-CM)* diagnoses and procedure codes

eTable 2. Postpartum mental health disorder and substance use disorder *International Classification of Diseases (ICD), 10th Revision, Clinical Modification* codes

eTable 3. Proportion of mental health disorder (MHD) related postpartum readmissions by individual characteristics at 42 days, 90 days, 180 days, and 365 days postpartum, in South Carolina, 2018-2021

eTable 4. Proportion of substance use disorder (SUD) related postpartum readmissions by individual characteristics at 42 days, 90 days, 180 days, and 365 days postpartum, in South Carolina, 2018-2021

eTable 5. Proportion of all-cause, mental health disorder (MHD) and substance used disorder (SUD)-related postpartum readmissions by timing across individual race, ethnicity, and residence location groups, in South Carolina 2018-2021

eTable 6. Hazard ratios of all causes, mental health disorder (MHD)-, and substance used disorder (SUD)-related postpartum readmissions by individual characteristics in South Carolina

eTable 7. Proportion of individuals who experienced multiple postpartum readmissions (PPR) within 1 year of birth

eFigure. Study population flowchart

This supplementary material has been provided by the authors to give readers additional information about their work.

© 2025 Tucker CM et al. *JAMA Network Open*.

eTable 1. Postpartum readmission *International Classification of Disease, Tenth Revision, Clinical Modification (ICD-10-CM)* diagnoses and procedure codes

Readmission Diagnoses	ICD-10 codes
**Diagnostic code**	
Acute Cardiovascular Disease (including cardiomyopathy, heart failure, myocardial infarction)	I21.01, I21.02, I21.09, I21.11, I21.19, I21.21, I21.29, I21.3, I22.0, I22.1, I22.2, I22.8, I20.0, I24.0, I24.8, I24.9, I25.110, I25.700, I25.710, I25.720, I25.730, I25.750, I25.760, I25.790, I50.21, I50.23, I50.31, I50.33, I50.41, I50.42, I50.43, O90.3, R57.0
Acute Cerebrovascular Disease (including stroke and intracranial hemorrhage)	I60-I63, I65-I69, G45-G46
Anesthesia Complications	O740-O749, O891-O899, O8909, O8901
Appendicitis	K352-K353, K3580, K3589, K36-K38
Sepsis	A40-A41, O85-O86, R6521, T8112
Gallbladder Disease	K8000-K8001, K8010-K8013, K8018-K8021, K8030-K8037, K8040-K8047, K8050-K8051, K8060-K8067, K8070-K8071, K8080-K8081, A5274, K810-K812, K819-K824, K828-K829, K915, K830-K835, K838-K839, K87
Non-specific Postpartum Diagnosis	O2663, O2673, O9953, O9963, O9973, O9A13, O9A23, O9A33, O9A43, O9A53, O9089, O99355, O99285, O99825, O253
Hemorrhage and/or Retained Products of Conception	D6951, D6959, O9903, O9913, I9742, I9762, K9161-K9162, N9961, G9731-G9732, G9751-G9752, J9561-J9562, E3601-E3602, H9521-H9522, H9541-H9542, L7601-L7602, L7621-L7622, D7801-D7802, D7821-D7822, N9962, O720-O723, O730-O731, O43211-O43213, O43219, O43221-O43223, O43229, O43231-O43233, O43239, I97410-I97411, I97418, I97610-I97611, I97618, K91840-K91841, N99820-N99821, M96810-M96811, M96830-M96831, J95830-J95831, H59111-H59112, H59121-H59122, H59311-H59313, H59321-H59323, H59119, H59129, H59319, H59329
Hypertensive Disorder	I10, I110, I119, I120, I129, I130, I132, I150-I152, I158-I159, O111-O113, O119, O131-O133, O139, O151, O152, O159, O161-O163, O169, I1310, I1311, O1002, O1003, O1012, O1013, O1022, O1023, O1032, O1033, O1042, O1043, O1092, O1093, O1412, O1413, O1422, O1423, O1500,

© 2025 Tucker CM et al. *JAMA Network Open*.

	O1502, O1503, O10011-O10013, O10019, O10111-O10113, O10211-O10213, O10219, O10311-O10313, O10319, O10411-O10413, O10419, O10911-O10913, O10919
Mastitis, Breast Abscess	O91011-O91013, O91019, O91111-O91113, O91119, O91211-O91213, O91219, O9102, O9103, O9112, O9113, O9122, O9123, N61
Pancreatitis	B252, K850-K853, K858-K863, K868-K869, K87
Psychiatric Disease (including substance abuse)	F01-F25, F28-F34, F39-F44, F48, F50-F55, F59-F60, F63, F68-F69, F84, F93, F94, O906, Z8651, Z8659, O99320-O99325, O99310-O99315, O99340-O99345, O99441-O99445, O355XX0-O355XX5, O355XX9
Thrombotic Event	I26, I82, O22, O87, O88
Upper Respiratory Tract Infection	J01-J18, J31-J42, J44, A221, A481, B250, B440, A3701, A3711, A3781, A3791, B7781
Urinary Tract Infection (including pyelonephritis)	N10, N11, N12, N16, N30, O23, N136, N159, N151, N390, N2884, N2885,
	N2886, A5601, O8611, O8613, O8619, O8620, O8621, O8622, O8629
Uterine Infection	N70, N71, N73, N74, O85, A5611, O8612, O8681, O8689
Wound Infection and/or Breakdown	K65, K66, K67, K68, N72, N76, N77, N739, N750, N751, N758, N759, O860, O900, O901, O902, A5602, T8130XA, T8131XA, T8132XA, T8133XA, T8189XA, T814XXA
**Procedure Code**	
Dilation and Curettage	10D17ZZ, 10D18ZZ, 0UDB7ZX, 0UDB7ZZ, 0UDB8ZX, 0UDB8ZZ
Hysterectomy	0UT90ZZ, 0UT94ZZ, 0UTC4ZZ, 0UT9FZZ, 0UT97ZZ, 0UT98ZZ, 0UTC7ZZ, 0UTC8ZZ, 0UT44ZZ", "0UT40ZZ, 0UTC0ZZ, 0UT47ZZ, 0UT48ZZ, 0DTN0ZZ, 0DTP0ZZ, 0TTB0ZZ, 0TTD0ZZ, 0UT20ZZ, 0UT70ZZ, 0UTG0ZZ, 0UT94ZL, 0UT90ZL, 0UT97ZL, 0UT98ZL, 0UT9FZL
Laparotomy	0DJ00ZZ, 0DJ60ZZ, 0DJD0ZZ, 0DJU0ZZ, 0DJW0ZZ, 0WJG0ZZ, 0WJJ0ZZ, 0WJP0ZZ, 0WJR0ZZ, 0W3G0ZZ, 0W3H0ZZ, 0W3P0ZZ, 0WJH0ZZ, 0D9S00Z, 0D9S0ZZ, 0D9T00Z, 0D9T0ZZ, 0D9V00Z, 0D9V0ZZ, 0D9W00Z, 0D9W0ZZ, 0W9G00Z, 0W9G0ZZ, 0WCJ0ZZ, 0WCP0ZZ, 0WCR0ZZ

© 2025 Tucker CM et al. *JAMA Network Open*.

Transfusion	30233H0, 30233N0, 30243H0, 30243N0, 30253H0, 30253N0, 30263H0, 30263N0, 30233H1, 30243H1, 30253H1, 30263H1, 30233W0, 30243W0, 30253H0, 30253N0, 30253W0, 30263H0, 30263N0, 30263W0, 30253H1, 30263H1, 30233N1, 30233P1, 30243N1, 30243P1, 30253N1, 30253P1, 30263N1, 30263P1, 30233R1, 30243R1, 30253R1, 30263R1, 30233T1, 30233V1, 30233W1, 30243T1, 30243V1, 30243W1, 30253T1, 30253V1, 30253W1, 30263T1, 30263V1, 30263W1, 30233J1, 30233K1, 30233L1, 30233M1, 30243J1, 30243K1, 30243L1, 30243M1, 30253J1, 30253K1, 30253L1, 30253M1, 30263J1, 30263K1, 30263L1, 30263M1, 3E033GC, 3E043GC, 3E053GC, 3E063GC, 30233Q1, 30243Q1, 30253Q1, 30263Q1

eTable 2. Postpartum mental health disorder and substance use disorder *International Classification of Diseases (ICD), 10th Revision, Clinical Modification* codes

	**Condition**	**ICD-10 Codes**
Mental Health Disorder	Mental and behavioral disorders due to psychoactive substance use	F10, F11, F12, F13, F14, F15, F16, F17, F18, F19, O99.32
	Other substance use disorders	T40, T41, T43.6, G31.2, G62.1, I42.6, K29.2, G70, O35.4, O35.5
	Psychosis	F20, F21, F22, F23, F24, F25, F26, F27, F28, F29, F53.1
	Mood disorders (including depression)	F30, F31, F32, F33, F34, F35, O90.6, F53.0
	Anxiety and stress-related disorders	F40, F41, F42, F43, F44, F45, F48
	Sleep disorders (not due to substance or known physiological condition)	F51.0, G47.0
	Other mental disorders associated with postpartum period	O99.345, Z86.59
Substance use disorder	Opioids	F11, T40.1, T40.2, T40.3, T40.4, T40.6
	Cocaine	F14, T40.5
	Other stimulants	F15, T43.6
	All other substances	F10, F12, F13, F16, F17, F18, F19, G31.2, G62.1, I42.6, K29.2, G70, O35.4, O35.5, O99.32, T40.7, T40.8, T40.9, T41

© 2025 Tucker CM et al. *JAMA Network Open*.

eTable 3. Proportion of mental health disorder (MHD) related postpartum readmissions by individual characteristics at 42 days, 90 days, 180 days, and 365 days postpartum, in South Carolina, 2018-2021, *N* (%) [a,b]

**Individual Characteristics**	**Overall N = 190,645**	42 days		90 days		180 days		365 days	
		N = 807 (0.4%)	***p value***	N = 1,082 (0.6)	***p value***	N = 1,521 (0.8%)	***p value***	N = 2,896 (1.5%)	***p value***
**Race or Ethnicity**			<0.001		<0.001		<0.001		<0.001
Non-Hispanic Black	58,859 (30.9%)	288 (0.5%)		380 (0.6%)		541 (0.9%)		1,006 (1.7%)	
Non-Hispanic White	108,812 (57.1%)	476 (0.4%)		643 (0.6%)		893 (0.8%)		1,720 (1.6%)	
Hispanic	9,400 (4.9%)	16 (0.2%)		19 (0.2%)		29 (0.3%)		54 (0.6%)	
Others[c]	13,574 (7.1%)	27 (0.2%)		40 (0.3%)		58 (0.4%)		116 (0.9%)	
**Residence Rurality**			0.6		0.08		0.014		0.002
Rural	27,961 (14.7%)	123 (0.4%)		179 (0.6%)		257 (0.9%)		483 (1.7%)	
Urban	162,684 (85.3%)	684 (0.4%)		903 (0.6%)		1,264 (0.8%)		2,413 (1.5%)	
**Individual Age**			<0.001		0.004		0.077		<0.001
<20	11,655 (6.1%)	33 (0.3%)		51 (0.4%)		85 (0.7%)		225 (1.9%)	
20-24	42,914 (22.5%)	146 (0.3%)		234 (0.5%)		368 (0.9%)		771 (1.8%)	
25-29	57,921 (30.4%)	228 (0.4%)		303 (0.5%)		442 (0.8%)		866 (1.5%)	
30-34	49,997 (26.2%)	228 (0.5%)		297 (0.6%)		374 (0.7%)		650 (1.3%)	
≥35	28,158 (14.8%)	172 (0.6%)		197 (0.7%)		252 (0.9%)		384 (1.4%)	
**Education Attainment**			<0.001		<0.001		<0.001		<0.001
Bachelor's degree	34,976 (18.3%)	88 (0.3%)		101 (0.3%)		132 (0.4%)		210 (0.6%)	

© 2025 Tucker CM et al. *JAMA Network Open*.

Graduate school	19,707 (10.3%)	52 (0.3%)		63 (0.3%)		82 (0.4%)		108 (0.5%)	
High school diploma	49,102 (25.8%)	228 (0.5%)		327 (0.7%)		465 (0.9%)		929 (1.9%)	
No high school diploma	24,072 (12.6%)	136 (0.6%)		188 (0.8%)		280 (1.2%)		653 (2.7%)	
Some college	62,788 (32.9%)	303 (0.5%)		403 (0.6%)		562 (0.9%)		996 (1.6%)	
**Primary Payer at Birth**			<0.001		<0.001		<0.001		<0.001
Private	76,651 (40.2%)	238 (0.3%)		302 (0.4%)		401 (0.5%)		657 (0.9%)	
Medicaid	88,240 (46.3%)	481 (0.5%)		664 (0.8%)		942 (1.1%)		1,905 (2.2%)	
Other Public	21,998 (11.5%)	80 (0.4%)		106 (0.5%)		161 (0.7%)		291 (1.3%)	
Uninsured	3,756 (2.0%)	8 (0.2%)		10 (0.3%)		17 (0.5%)		43 (1.1%)	
**Cesarean Delivery**			<0.001		<0.001		<0.001		<0.001
Yes	63,389 (33.2%)	415 (0.7%)		523 (0.8%)		698 (1.1%)		1,175 (1.9%)	
**Trimester Prenatal Care Began**			0.043		<0.001		<0.001		<0.001
First Trimester	144,710 (75.9%)	583 (0.4%)		768 (0.5%)		1,082 (0.7%)		2,039 (1.4%)	
Second Trimester	35,407 (18.6%)	176 (0.5%)		250 (0.7%)		336 (0.9%)		640 (1.8%)	
Third Trimester	10,528 (5.5%)	48 (0.5%)		64 (0.6%)		103 (1.0%)		217 (2.1%)	
**Kotelchuck**			<0.001		<0.001		<0.001		<0.001
Inadequate	32,959 (16.9%)	179 (0.5%)		246 (0.7%)		358 (1.1%)		713 (2.2%)	
Intermediate	10,125 (5.2%)	44 (0.4%)		62 (0.6%)		92 (0.9%)		174 (1.7%)	
Adequate	53,864 (28.3%)	147 (0.3%)		210 (0.4%)		296 (0.5%)		585 (1.1%)	

© 2025 Tucker CM et al. *JAMA Network Open*.

Adequate Plus	94,658 (49.7%)	447 (0.5%)		576 (0.6%)		789 (0.8%)		1,454 (1.5%)	
**Parity**			<0.001		<0.001		<0.001		<0.001
Primary Birth	75,209 (39.4%)	288 (0.4%)		382 (0.5%)		531 (0.7%)		1,042 (1.4%)	
One Previous Live Birth	60,434 (31.7%)	227 (0.4%)		309 (0.5%)		450 (0.7%)		822 (1.4%)	
At least Two Previous Live Birth	55,002 (28.9%)	292 (0.5%)		391 (0.7%)		540 (1.0%)		1,032 (1.9%)	
**Childbirth Hospitalization Length of Stay**			<0.001		<0.001		<0.001		<0.001
0-1 Days	19,261 (10.1%)	72 (0.4%)		96 (0.5%)		132 (0.7%)		246 (1.3%)	
2-3 Days	145,666 (76.4%)	485 (0.3%)		671 (0.5%)		964 (0.7%)		1,961 (1.3%)	
4+ Days	25,718 (13.5%)	250 (1.0%)		315 (1.2%)		425 (1.7%)		689 (2.7%)	
**Year of Birth**			0.041		0.021		0.01		0.02
2018	47,607 (25.0%)	207 (0.4%)		276 (0.6%)		403 (0.8%)		775 (1.6%)	
2019	48,714 (25.6%)	192 (0.4%)		262 (0.5%)		369 (0.8%)		709 (1.5%)	
2020	47,684 (25.0%)	180 (0.4%)		241 (0.5%)		338 (0.7%)		674 (1.4%)	
2021	46,640 (24.5%)	228 (0.5%)		303 (0.6%)		411 (0.9%)		738 (1.6%)	
**Obstetric Comorbidity Index Score**			<0.001		<0.001		<0.001		<0.001
0	70,484 (37.0%)	81 (0.1%)		126 (0.2%)		223 (0.3%)		488 (0.7%)	
1-8	59,837 (31.4%)	264 (0.4%)		361 (0.6%)		503 (0.8%)		984 (1.6%)	
9-14	40,508 (21.2%)	180 (0.4%)		254 (0.6%)		334 (0.8%)		678 (1.7%)	
15+	19,816 (10.4%)	282 (1.4%)		341 (1.7%)		461 (2.3%)		746 (3.8%)	

© 2025 Tucker CM et al. *JAMA Network Open*.

a. *p* values were calculated to compare individual characteristics with MHD-related PPR up to 365 days postpartum using Pearson's Chi-square tests.
b. Row percentages presented.
c. Other race and ethnicity groups included American Indian or Alaska Native, Asian, Native Hawaiian, Pacific Islander, and/or multiple races.

© 2025 Tucker CM et al. *JAMA Network Open*.

eTable 4. Proportion of substance use disorder (SUD) related postpartum readmissions by individual characteristics at 42 days, 90 days, 180 days, and 365 days postpartum, in South Carolina, 2018-2021, *N* (%) [a,b]

Individual Characteristics	N = 190,645	42 days		90 days		180 days		365 days	
		N = 350 (0.2%)	*p value*	N = 494 (0.3%)	*p value*	N = 757 (0.4%)	*p value*	N = 1,601 (0.8%)	*p value*
**Race and ethnicity**			<0.001		<0.001		<0.001		<0.001
Non-Hispanic Black	58,859 (30.9%)	137 (0.2%)		184 (0.3%)		280 (0.5%)		573 (1.0%)	
Non-Hispanic White	108,812 (57.1%)	204 (0.2%)		292 (0.3%)		445 (0.4%)		958 (0.9%)	
Hispanic	9,400 (4.9%)	5 (0.1%)		7 (<0.1%)		10 (0.1%)		19 (0.2%)	
Others[C]	13,574 (7.1%)	4 (0.0%)		11 (<0.1%)		22 (0.2%)		51 (0.4%)	
**Residence Rurality**			0.005		<0.001		<0.001		<0.001
Rural	27,961 (14.7%)	70 (0.3%)		103 (0.4%)		149 (0.5%)		311 (1.1%)	
Urban	162,684 (85.3%)	280 (0.2%)		391 (0.2%)		608 (0.4%)		1,290 (0.8%)	
**Individual Age**			<0.001		0.04		0.2		<0.001
<20	11,655 (6.1%)	13 (0.1%)		17 (0.1%)		38 (0.3%)		117 (1.0%)	
20-24	42,914 (22.5%)	55 (0.1%)		103 (0.2%)		190 (0.4%)		454 (1.1%)	
25-29	57,921 (30.4%)	107 (0.2%)		153 (0.3%)		233 (0.4%)		508 (0.9%)	
30-34	49,997 (26.2%)	97 (0.2%)		132 (0.3%)		179 (0.4%)		332 (0.7%)	
≥35	28,158 (14.8%)	78 (0.3%)		89 (0.3%)		117 (0.4%)		190 (0.7%)	
**Education Attainment**			<0.001		<0.001		<0.001		<0.001
Bachelor's degree	34,976 (18.3%)	NR		13 (<0.1%)		22 (<0.1%)		37 (0.1%)	
Graduate school	19,707 (10.3%)	NR		NR		NR		11 (<0.1%)	
High school diploma	49,102 (25.8%)	121 (0.2%)		176 (0.4%)		264 (0.5%)		589 (1.2%)	

© 2025 Tucker CM et al. *JAMA Network Open*.

No high school diploma	24,072 (12.6%)	92 (0.4%)		129 (0.5%)		198 (0.8%)		461 (1.9%)	
Some college	62,788 (32.9%)	122 (0.2%)		168 (0.3%)		263 (0.4%)		503 (0.8%)	
**Primary Payer at Birth**			<0.001		<0.001		<0.001		<0.001
Private	76,651 (40.2%)	52 (0.1%)		80 (0.1%)		116 (0.2%)		225 (0.3%)	
Medicaid	88,240 (46.3%)	269 (0.3%)		374 (0.4%)		566 (0.6%)		1,222 (1.4%)	
Other Public	21,998 (11.5%)	26 (0.1%)		36 (0.2%)		66 (0.3%)		129 (0.6%)	
Uninsured	3,756 (2.0%)	3,756 (2.0%)		NR		NR		25 (0.7%)	
**Cesarean Delivery**			<0.001		<0.001		<0.001		<0.001
Yes	63,389 (33.2%)	184 (0.3%)		246 (0.4%)		352 (0.6%)		624 (1.0%)	
**Trimester Prenatal Care Began**			<0.001		<0.001		<0.001		<0.001
First Trimester	144,710 (75.9%)	216 (0.1%)		311 (0.2%)		488 (0.3%)		1,040 (0.7%)	
Second Trimester	35,407 (18.6%)	107 (0.3%)		147 (0.4%)		204 (0.6%)		407 (1.1%)	
Third Trimester	10,528 (5.5%)	27 (0.3%)		36 (0.3%)		65 (0.6%)		154 (1.5%)	
**Kotelchuck**			<0.001		<0.001		<0.001		<0.001
Inadequate	9,995 (5.2%)	18 (0.2%)		30 (0.3%)		52 (0.5%)		106 (1.1%)	
Intermediate	32,128 (16.9%)	105 (0.3%)		138 (0.4%)		212 (0.7%)		469 (1.5%)	
Adequate	53,864 (28.3%)	69 (0.1%)		104 (0.2%)		159 (0.3%)		318 (0.6%)	
Adequate Plus	94,658 (49.7%)	158 (0.2%)		222 (0.2%)		334 (0.4%)		708 (0.7%)	
**Parity**			<0.001		<0.001		<0.001		<0.001
Primary Birth	75,209 (39.4%)	87 (0.1%)		126 (0.2%)		208 (0.3%)		485 (0.6%)	
One Previous Live Birth	60,434 (31.7%)	103 (0.2%)		143 (0.2%)		230 (0.4%)		443 (0.7%)	

© 2025 Tucker CM et al. *JAMA Network Open*.

At least Two Previous Live Birth	55,002 (28.9%)	160 (0.3%)		225 (0.4%)		319 (0.6%)		673 (1.2%)	
**Childbirth Hospitalization Length of Stay**			<0.001		<0.001		<0.001		<0.001
0-1 Days	19,261 (10.1%)	36 (0.2%)		51 (0.3%)		73 (0.4%)		153 (0.8%)	
2-3 Days	145,666 (76.4%)	226 (0.2%)		320 (0.2%)		499 (0.3%)		1,108 (0.8%)	
4+ Days	25,718 (13.5%)	88 (0.3%)		123 (0.5%)		185 (0.7%)		340 (1.3%)	
**Year of Birth**			0.2		0.2		<0.001		<0.001
2018	47,607 (25.0%)	104 (0.2%)		144 (0.3%)		238 (0.5%)		482 (1.0%)	
2019	48,714 (25.6%)	79 (0.2%)		116 (0.2%)		181 (0.4%)		405 (0.8%)	
2020	47,684 (25.0%)	81 (0.2%)		117 (0.2%)		170 (0.4%)		367 (0.8%)	
2021	46,640 (24.5%)	86 (0.2%)		117 (0.3%)		168 (0.4%)		347 (0.7%)	
**Obstetric Comorbidity Index Score**			<0.001		<0.001		<0.001		<0.001
0	70,484 (37.0%)	29 (0.0%)		48 (<0.1%)		106 (0.2%)		263 (0.4%)	
1-8	59,837 (31.4%)	108 (0.2%)		155 (0.3%)		244 (0.4%)		553 (0.9%)	
9-14	40,508 (21.2%)	75 (0.2%)		116 (0.3%)		158 (0.4%)		371 (0.9%)	
15+	19,816 (10.4%)	138 (0.7%)		175 (0.9%)		249 (1.3%)		414 (2.1%)	

a. *p* values were calculated to compare individual characteristics with SUD-related PPR up to 365 days postpartum using Pearson's Chi-square tests.
b. Row percentages presented.
c. Other race and ethnicity groups included American Indian or Alaska Native, Asian, Native Hawaiian, Pacific Islander, and/or multiple races.
d. NR: Not reportable due to data restrictions on sample size reporting.

© 2025 Tucker CM et al. *JAMA Network Open*.

eTable 5. Proportion of all-cause, mental health disorder (MHD) and substance used disorder (SUD)-related postpartum readmissions by timing across individual race, ethnicity, and residence location groups, in South Carolina 2018-2021, *N* (%) [a,b]

	Individual Characteristics	N = 190,645	42 days	*p* value	90 days	*p* value	180 days	*p* value	365 days	*p* value
**All-cause Diagnosis**			N = 3,500 (1.8%)		N = 4,155 (2.2%)		N = 5,038 (2.6%)		N = 8,976 (4.7%)	
	**Race or Ethnicity**			<0.001		<0.001		<0.001		<0.001
	Non- Hispanic Black	58,859 (30.9%)	1,579 (2.7%)		1,819 (3.1%)		2,154 (3.7%)		3,918 (6.7%)	
	Non-Hispanic White	108,812 (57.1%)	1,628 (1.5%)		1,951 (1.8%)		2,404 (2.2%)		4,218 (3.9%)	
	Hispanic	9,400 (4.9%)	125 (1.3%)		167 (1.8%)		206 (2.2%)		363 (3.9%)	
	Others[c]	13,574 (7.1%)	168 (1.2%)		218 (1.6%)		274 (2.0%)		477 (3.5%)	
	**Residence Rurality**			0.8		0.14		0.004		<0.001
	Rural	27,961 (14.7%)	518 (1.9%)		643 (2.3%)		810 (2.9%)		1,538 (5.5%)	
	Urban	162,684 (85.3%)	2,982 (1.8%)		3,512 (2.2%)		4,228 (2.6%)		7,438 (4.6%)	
**Mental Health Disorder**			N = 807 (0.4%)		N = 1,082 (0.6%)		N = 1,521 (0.8%)		N = 2,896 (1.5%)	
	**Race or Ethnicity**			<0.001		<0.001		<0.001		<0.001
	Non-Hispanic Black	58,859 (30.9%)	288 (0.5%)		380 (0.6%)		541 (0.9%)		1,006 (1.7%)	
	Non-Hispanic White	108,812 (57.1%)	476 (0.4%)		643 (0.6%)		893 (0.8%)		1,720 (1.6%)	
	Hispanic	9,400 (4.9%)	16 (0.2%)		19 (0.2%)		29 (0.3%)		54 (0.6%)	
	Others[c]	13,574 (7.1%)	27 (0.2%)		40 (0.3%)		58 (0.4%)		116 (0.9%)	
	**Residence Rurality**			0.6		0.072		0.012		<0.001
	Rural	27,961 (14.7%)	123 (0.4%)		179 (0.6%)		257 (0.9%)		483 (1.7%)	
	Urban	162,684 (85.3%)	684 (0.4%)		903 (0.6%)		1,264 (0.8%)		2,413 (1.5%)	

© 2025 Tucker CM et al. *JAMA Network Open*.

			N = 350[1]		N = 494[1]		N = 757[1]		N = 1,601[1]	
**Substance Use Disorder**	**Race or Ethnicity**			<0.001		<0.001		<0.001		<0.001
	Non-Hispanic Black	58,859 (30.9%)	137 (0.2%)		184 (0.3%)		280 (0.5%)		573 (1.0%)	
	Non-Hispanic White	108,812 (57.1%)	204 (0.2%)		292 (0.3%)		445 (0.4%)		958 (0.9%)	
	Hispanic	9,400 (4.9%)	NR		NR		NR		19 (0.2%)	
	Others[c]	13,574 (7.1%)	NR		11 (<0.1%)		22 (0.2%)		51 (0.4%)	
	**Residence Rurality**			0.005		<0.001		<0.001		<0.001
	Rural	27,961 (14.7%)	70 (0.3%)		103 (0.4%)		149 (0.5%)		311 (1.1%)	
	Urban	162,684 (85.3%)	280 (0.2%)		391 (0.2%)		608 (0.4%)		1,290 (0.8%)	

a. *p* values were calculated to compare individual characteristics with SUD-related PPR up to 365 days postpartum using Pearson’s Chi-square tests.
b. Row percentages presented.
c. Other race and ethnicity groups included American Indian or Alaska Native, Asian, Native Hawaiian, Pacific Islander, and/or multiple races.
d. NR: Not reportable due to data restrictions on sample size reporting.

© 2025 Tucker CM et al. *JAMA Network Open*.

eTable 6. Hazard ratios of all causes, mental health disorder (MHD)-, and substance used disorder (SUD)-related postpartum readmissions by individual characteristics in South Carolina, 2018-2021 [a,b]

Individual Characteristics	All-cause				Mental Health				Substance Use			
	Adjusted Model	Crude Model	Crude Model with Interaction	Adjusted Model with Interaction	Adjusted Model	Crude Model	Crude Model with Interaction	Adjusted Model with Interaction	Adjusted Model	Crude Model	Crude Model with Interaction	Adjusted Model with Interaction
**Race or Ethnicity**												
Non-Hispanic Black	1.345 (1.285-1.408)	1.742 (1.668-1.820)	1.819 (1.733-1.908)	1.382 (1.314-1.454)	0.734 (0.677-0.796)	1.082 (1.001-1.170)	1.131 (1.038-1.233)	0.745 (0.682-0.815)	0.671 (0.603-0.746)	1.106 (0.998-1.227)	1.144 (1.018-1.285)	0.673 (0.597-0.758)
Hispanic	0.789 (0.707-0.881)	0.996 (0.894-1.108)	1.066 (0.954-1.190)	0.827 (0.738-0.926)	0.229 (0.174-0.301)	0.362 (0.276-0.474)	0.390 (0.296-0.515)	0.239 (0.181-0.317)	0.112 (0.071-0.176)	0.229 (0.145-0.360)	0.250 (0.157-0.399)	0.115 (0.072-0.184)
Others[c]	0.842 (0.765-0.926)	0.905 (0.823-0.995)	0.911 (0.823-1.008)	0.841 (0.759-0.931)	0.455 (0.377-0.551)	0.539 (0.446-0.650)	0.542 (0.444-0.663)	0.454 (0.371-0.556)	0.312 (0.235-0.415)	0.426 (0.321-0.564)	0.423 (0.311-0.574)	0.303 (0.222-0.413)
**Residence Rurality**												
Rural	1.059 (1.002-1.120)	1.206 (1.142-1.274)	1.304 (1.201-1.416)	1.152 (1.060-1.251)	1.020 (0.924-1.126)	1.166 (1.057-1.286)	1.270 (1.116-1.446)	1.058 (0.929-1.205)	1.142 (1.008-1.294)	1.405 (1.241-1.590)	1.497 (1.270-1.765)	1.145 (0.971-1.351)
**Individual Age**												
<20	1.023 (0.939-1.114)	1.158 (1.069-1.256)		1.024 (0.940-1.114)	0.907 (0.776-1.060)	1.074 (0.926-1.246)		0.907 (0.776-1.060)	0.754 (0.609-0.934)	0.948 (0.774-1.162)		0.754 (0.609-0.934)
25-29	0.860 (0.811-0.911)	0.750 (0.710-0.793)		0.860 (0.811-0.912)	0.997 (0.901-1.105)	0.832 (0.755-0.916)		0.997 (0.901-1.105)	1.071 (0.937-1.223)	0.829 (0.730-0.941)		1.070 (0.937-1.223)
30-34	0.890 (0.833-0.951)	0.691 (0.651-0.733)		0.891 (0.834-0.952)	1.019 (0.907-1.144)	0.723 (0.651-0.802)		1.019 (0.908-1.144)	1.067 (0.915-1.246)	0.627 (0.544-0.722)		1.068 (0.915-1.246)
≥35	0.928 (0.859-1.004)	0.749 (0.700-0.803)		0.930 (0.860-1.006)	1.031 (0.900-1.182)	0.759 (0.672-0.858)		1.032 (0.900-1.183)	1.091 (0.907-1.312)	0.637 (0.538-0.755)		1.092 (0.908-1.313)

© 2025 Tucker CM et al. *JAMA Network Open*.

**Education Attainment**												
Bachelor's degree	0.632 (0.581-0.687)	0.448 (0.416-0.483)		0.634 (0.583-0.689)	0.424 (0.360-0.500)	0.316 (0.272-0.367)		0.425 (0.360-0.501)	0.136 (0.097-0.193)	0.088 (0.063-0.122)		0.136 (0.097-0.193)
Graduate school	0.614 (0.552-0.684)	0.423 (0.384-0.465)		0.616 (0.553-0.685)	0.408 (0.329-0.507)	0.288 (0.236-0.352)		0.409 (0.329-0.507)	0.079 (0.043-0.145)	0.046 (0.025-0.084)		0.079 (0.043-0.145)
No high school diploma	1.160 (1.088-1.236)	1.142 (1.075-1.215)		1.158 (1.087-1.235)	1.440 (1.298-1.597)	1.438 (1.301-1.589)		1.439 (1.297-1.596)	1.573 (1.387-1.785)	1.602 (1.418-1.809)		1.573 (1.387-1.785)
Some college	0.928 (0.880-0.978)	0.848 (0.806-0.892)		0.927 (0.879-0.977)	0.880 (0.801-0.965)	0.838 (0.766-0.916)		0.879 (0.801-0.965)	0.722 (0.638-0.816)	0.667 (0.592-0.751)		0.721 (0.638-0.816)
**Insurance Type**												
Medicaid	1.284 (1.214-1.358)	1.856 (1.771-1.946)		1.283 (1.214-1.357)	1.667 (1.504-1.847)	2.533 (2.318-2.768)		1.666 (1.503-1.846)	2.244 (1.923-2.619)	4.743 (4.114-5.468)		2.244 (1.923-2.619)
Other Public	1.156 (1.072-1.248)	1.267 (1.175-1.366)		1.160 (1.075-1.252)	1.386 (1.206-1.594)	1.547 (1.347-1.775)		1.389 (1.208-1.597)	1.550 (1.247-1.927)	2.001 (1.611-2.484)		1.551 (1.247-1.929)
Uninsured	0.933 (0.778-1.120)	0.990 (0.827-1.186)		0.933 (0.778-1.120)	1.187 (0.869-1.622)	1.336 (0.981-1.819)		1.188 (0.869-1.622)	1.517 (0.999-2.304)	2.271 (1.502-3.432)		1.517 (0.999-2.304)
**Cesarean Delivery**												
Yes	1.074 (1.025-1.125)	1.311 (1.257-1.368)		1.074 (1.025-1.125)	1.009 (0.930-1.093)	1.376 (1.278-1.482)		1.008 (0.930-1.093)	0.994 (0.891-1.108)	1.285 (1.162-1.421)		0.993 (0.891-1.108)
**Kotelchuck**												
Intermediate	0.960 (0.870-1.060)	0.874 (0.791-0.965)		0.961 (0.870-1.062)	0.900 (0.761-1.064)	0.800 (0.677-0.946)		0.900 (0.761-1.065)	0.839 (0.680-1.037)	0.725 (0.587-0.895)		0.840 (0.680-1.037)
Adequate	0.831 (0.779-0.887)	0.635 (0.596-0.677)		0.831 (0.779-0.887)	0.713 (0.637-0.798)	0.506 (0.453-0.565)		0.713 (0.637-0.797)	0.645 (0.558-0.745)	0.403 (0.349-0.464)		0.645 (0.558-0.745)

© 2025 Tucker CM et al. *JAMA Network Open*.

Adequate Plus	0.983 (0.930-1.039)	0.871 (0.825-0.919)		0.982 (0.929-1.038)	0.816 (0.743-0.896)	0.718 (0.655-0.786)		0.816 (0.743-0.895)	0.656 (0.582-0.739)	0.511 (0.454-0.574)		0.656 (0.582-0.739)
**Parity**												
One Previous Live Birth	0.900 (0.852-0.951)	0.881 (0.837-0.927)		0.900 (0.852-0.951)	0.929 (0.842-1.025)	0.982 (0.896-1.076)		0.929 (0.842-1.025)	0.999 (0.870-1.146)	1.138 (1.000-1.294)		0.999 (0.870-1.147)
At least Two Previous Live Birth	0.954 (0.899-1.013)	1.101 (1.049-1.157)		0.954 (0.899-1.013)	0.999 (0.900-1.108)	1.358 (1.246-1.480)		0.999 (0.900-1.108)	1.154 (1.002-1.329)	1.903 (1.694-2.139)		1.154 (1.002-1.329)
**Childbirth Hospitalization Length of Stay**												
0-1 Days	1.193 (1.105-1.288)	0.952 (0.884-1.025)		1.194 (1.106-1.289)	1.183 (1.032-1.356)	0.949 (0.831-1.083)		1.183 (1.033-1.357)	1.301 (1.093-1.548)	1.045 (0.882-1.237)		1.301 (1.093-1.548)
4+ Days	1.367 (1.293-1.445)	1.813 (1.723-1.908)		1.366 (1.292-1.444)	1.430 (1.301-1.572)	2.008 (1.841-2.190)		1.429 (1.300-1.571)	1.284 (1.126-1.464)	1.744 (1.545-1.970)		1.283 (1.125-1.463)
**Year of Birth**												
2019	0.916 (0.865-0.971)	0.918 (0.866-0.973)		0.916 (0.865-0.971)	0.901 (0.814-0.997)	0.893 (0.807-0.989)		0.901 (0.813-0.997)	0.838 (0.735-0.957)	0.820 (0.719-0.936)		0.838 (0.735-0.957)
2020	0.893 (0.842-0.947)	0.906 (0.855-0.961)		0.893 (0.842-0.946)	0.862 (0.777-0.955)	0.867 (0.782-0.961)		0.861 (0.777-0.955)	0.763 (0.666-0.874)	0.759 (0.663-0.870)		0.763 (0.666-0.874)
2021	0.970 (0.915-1.028)	0.970 (0.915-1.027)		0.969 (0.915-1.027)	0.982 (0.888-1.087)	0.972 (0.879-1.075)		0.982 (0.888-1.087)	0.767 (0.668-0.881)	0.734 (0.639-0.842)		0.767 (0.668-0.881)
**Obstetric Comorbidity Index Score**												
1-8	1.420 (1.339-1.505)	1.502 (1.420-1.588)		1.419 (1.338-1.504)	2.152 (1.924-2.407)	2.387 (2.142-2.661)		2.151 (1.923-2.406)	2.121 (1.823-2.468)	2.484 (2.145-2.877)		2.121 (1.823-2.468)

© 2025 Tucker CM et al. *JAMA Network Open*.

9-14	1.551 (1.460-1.649)	1.811 (1.707-1.921)		1.549 (1.457-1.646)	2.091 (1.856-2.357)	2.430 (2.163-2.730)		2.089 (1.854-2.354)	1.989 (1.692-2.339)	2.462 (2.102-2.883)		1.989 (1.691-2.338)
15+	2.352 (2.194-2.521)	3.062 (2.877-3.259)		2.347 (2.189-2.515)	4.168 (3.676-4.725)	5.540 (4.943-6.210)		4.163 (3.672-4.720)	3.984 (3.364-4.719)	5.659 (4.849-6.605)		3.983 (3.363-4.717)
**Interaction term**												
Non-Hispanic Black * Rural			0.750 (0.669-0.840)	0.863 (0.770-0.967)			0.748 (0.611-0.917)	0.922 (0.752-1.130)			0.763 (0.591-0.986)	0.986 (0.763-1.274)
Hispanic * Rural			0.477 (0.295-0.772)	0.551 (0.341-0.892)			0.364 (0.088-1.502)	0.466 (0.113-1.924)			0.446 (0.059-3.364)	0.621 (0.082-4.686)
Others* Rural			1.018 (0.766-1.354)	1.038 (0.781-1.380)			1.012 (0.571-1.797)	1.037 (0.584-1.841)			1.164 (0.538-2.521)	1.239 (0.572-2.683)

a. The adjusted model was controlling for all covariates listed.
b. The crude models were fitted respectively without accounting for other confounders.
c. Other race and ethnicity groups included American Indian or Alaska Native, Asian, Native Hawaiian, Pacific Islander, and/or multiple races.

© 2025 Tucker CM et al. *JAMA Network Open*.

eTable 7. Proportion of individuals who experienced multiple postpartum readmissions (PPR) within 1 year of birth

#All-cause Diagnosis PPRs for each Individual	# of Individuals
1	7,736 (86.2%)
2	919 (10.2%)
3	178 (2.0%)
4	77 (0.9%)
5	22 (0.2%)
≥6	44 (0.5%)
	Total: 8,976
#SUD-related Diagnosis PPR for each Individual	#Mother
1	1,552 (96.9%)
2	46 (2.9%)
3	3 (0.2%)
	Total: 1,601
#MHD-related Diagnosis PPR for each Individual	#Mother
1	2,812 (97.1%)
2	75 (2.6%)
3	4 (0.1%)
4	5 (0.2%)
	Total: 2,896

© 2025 Tucker CM et al. *JAMA Network Open*.

eFigure. Study population flowchart

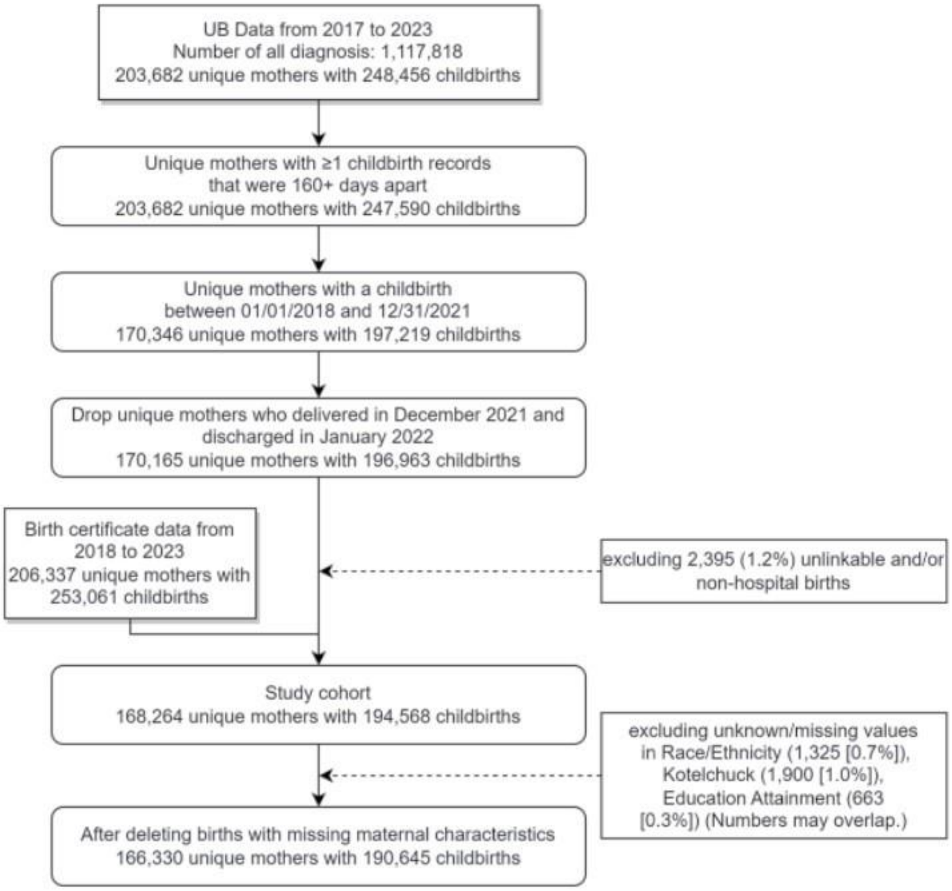

Supplement: Supplement 1. — eTable 1. Postpartum readmission International Classification of Disease, Tenth Revision, Clinical Modification (ICD-10-CM) diagnoses and procedure codes eTable 2. Postpartum mental health disorder and substance use disorder International Classification of Diseases (ICD), 10th Revision, Clinical Modification codes eTable 3. Proportion of mental health disorder (MHD) related postpartum readmissions by individual characteristics at 42 days, 90 days, 180 days, and 365 days postpartum, in South Carolina, 2018-2021 eTable 4. Proportion of substance use disorder (SUD) related postpartum readmissions by individual characteristics at 42 days, 90 days, 180 days, and 365 days postpartum, in South Carolina, 2018-2021 eTable 5. Proportion of all-cause, mental health disorder (MHD) and substance used disorder (SUD)-related postpartum readmissions by timing across individual race, ethnicity, and residence location groups, in South Carolina 2018-2021 eTable 6. Hazard ratios of all causes, mental health disorder (MHD)-, and substance used disorder (SUD)-related postpartum readmissions by individual characteristics in South Carolina eTable 7. Proportion of individuals who experienced multiple postpartum readmissions (PPR) within 1 year of birth eFigure. Study population flowchart [file jamanetwopen-e2547455-s001.pdf]
