# Supplementary material for: Variations in Postpartum Readmission by Individual Race, Ethnicity, and Rurality in South Carolina
Source: JAMA Netw Open. 2025 Dec 8;8(12):e2547455. doi: 10.1001/jamanetworkopen.2025.47455 (PMC12687097; doi:10.1001/jamanetworkopen.2025.47455)
Supplement: Supplement 2. — Data Sharing Statement [file jamanetwopen-e2547455-s002.pdf]

Data Sharing Statement

Tucker. Variations in Postpartum Readmission by Individual Race, Ethnicity, and Rurality in South Carolina. *JAMA Netw Open*. Published December 08, 2025.
doi:10.1001/jamanetworkopen.2025.47455

Data
Data available: No

Additional Information
Explanation for why data not available: The study data are not publicly available due to data use agreement restrictions. Researchers who wish to access the study data must contact the South Carolina Revenue and Fiscal Affairs Office (SC RFA) to request permission. Data will be shared in accordance with SC RFA policies and applicable data use agreements.
